# Tetramethylpyrazine protects mitochondrial function by up-regulation of TFAM and inhibition of neuronal apoptosis in a rat model of surgical brain injury

**DOI:** 10.22038/IJBMS.2023.72947.15862

**Published:** 2024

**Authors:** Chaoyu Wang, Yaqian Huang, Yating Gong, Muyao Wu, Lei Jiang, Baoqi Dang

**Affiliations:** 1 Department of Rehabilitation, Zhangjiagang TCM Hospital Affiliated to Nanjing University of Chinese Medicine, Suzhou, China; # These authors contributed eqully to this work

## Abstract

**Objective(s):**

Mitochondrial dysfunction caused by mitochondrial DNA (mtDNA) damage and mutation is widely accepted as one of the pathological processes of neurodegenerative diseases. As an mtDNA binding protein, mitochondrial transcription factor A (TFAM) maintains the integrity of mtDNA through transcription, replication, nucleoid formation, damage perception, and DNA repair. In recent works, the overexpression of TFAM increased the mtDNA copy count, promoted mitochondrial function, and improved the neurological dysfunction of neurodegenerative diseases. The role of TFAM in neurodegenerative diseases has been well explained. However, the role of TFAM after surgical brain injury (SBI) has not been studied. In this work, we aimed to study the role of TFAM in the brain after SBI and its mechanism of action.

**Materials and Methods:**

One hour after the occurrence of SBI, tetramethylpyrazine (TMP) was injected into the abdominal cavity of rats, and the brain was collected 48 hr later for testing. The evaluation included neurobehavioral function test, brain water content measurement, immunofluorescence, western blot, TUNEL staining, FJC staining, ROS test, and ATP test.

**Results:**

After SBI, the content of TFAM on the ipsilateral side increased and reached a peak at about 48 hr. After intraperitoneal injection of TMP in rats, 48 hr after SBI, the concentration of TFAM, Bcl-2, and adenosine triphosphate (ATP) increased; the content of caspase-3, reactive oxygen species (ROS), and cerebral edema decreased; and the nerve function significantly improved.

**Conclusion:**

TMP inhibited cell apoptosis after SBI in rats by up-regulating TFAM and protecting brain tissues.

## Introduction

Every year, 13.8 million people worldwide need neurosurgical treatment due to factors such as traumatic brain injury, cerebrovascular accidents, hydrocephalus, brain tumors, and vascular abnormalities (1). Despite the continuous development of modern science and surgical techniques, neurosurgery, including incision, retraction, and electrocautery, can inevitably damage the brain tissue surrounding the operation while treating the disease. This type of brain injury is known as surgical brain injury (SBI) (2, 3). Brain edema, oxidative stress, mitochondrial dysfunction, and nerve cell death are some pathological changes occurring after SBI. These changes aggravate postoperative nerve injury and affect its prognosis (4-6). The specific pathogenesis of SBI remains to be further explored. In particular, neuronal damage after brain injury requires special attention and is the key to treatment.

Neuronal cells require more energy than other cells. Mitochondria are important organelles in eukaryotes and are involved in regulating cellular energy metabolism (7). Mitochondria are responsible for the production of ATP and oxygen-free radicals in cells. They are also the main source of energy for organisms. Neurons have high energy demands. Therefore, mitochondria are critical to regulating neuronal function (8, 9); mitochondria also play key roles in apoptosis and in producing ROS (10, 11). When mitochondrial dysfunction occurs after SBI, ATP production is reduced, and neuron damage is more serious (12). A growing body of data suggests that mitochondrial dysfunction can promote cell death through the mitochondrial apoptotic pathway (13). Mitochondrial dysfunction can lead to oxidative stress response and excessive production of ROS; mitochondrial outer membrane translocation leads to the opening of the mitochondrial permeability transition pore, which increases mitochondrial membrane permeability. As a result, mitochondrial cytochrome c is released from the mitochondrial intermembrane space into the cytoplasm. Cytochrome c activates the mitochondria-dependent apoptotic pathways and also continuously initiates signal cascades (14-16), including Caspase-3, which ultimately leads to cell apoptosis (17, 18). Studies have shown that mitochondrial dysfunction plays a key role in the development of secondary damage induced by subarachnoid hemorrhage (19). ROS production is increased by brain injury, leading to mitochondrial damage, a decrease in ATP synthesis, and the activation of the endogenous apoptotic pathways. Neuronal apoptosis increases and nerve function damage is aggravated. Therefore, in this work, we investigated the possibilities of mitochondrial dysfunction as a potential therapeutic target for SBI neuronal injury.

TFAM is a nuclear coding protein that is widely expressed in the liver, heart, kidney, brain, and skeletal muscles (20). In the nucleus, TFAM is synthesized, and then it is transported to the cell’s mitochondria through the HSP60-HSP70 complex to maintain mtDNA. Lon protease binds to HSP60 in the HSP60-HSP70 complex and promotes the release of TFAM (21). Mitochondria upstream of the mtDNA light chain and heavy chain promoters stimulate the transcription of mtDNA, increase the copy number of mtDNA, and help to stabilize mtDNA (22, 23). TFAM that is not bound to the HSP60-HSP70 complex will be degraded by Lon protease.

Tetramethylpyrazine (TMP) is an alkaloid that occurs naturally in chuanxiong rhizome, and it has been extracted and applied as a therapeutic due to its wide range of biological activities (24). In a recent study, a subarachnoid hemorrhage (SAH) animal model was treated with TMP, and the mitochondrial-dependent Caspase-3 apoptosis pathway was inhibited (25). Through direct interaction with TFAM, TMP stabilizes TFAM, leading to inhibition of the Lon protease pathway, a reduction in TFAM degradation, and an increase in mtDNA copy number (26). These interactions led us to investigate whether TMP can mitigate neuronal damage after the occurrence of SBI. Therefore, we established an SBI rat model and intervened by intraperitoneal injection of TMP to observe whether TMP could increase TFAM content. Can the increase of TFAM content play a role in protecting mitochondrial function, thereby reduce the production of ROS and improve neurological function?

## Materials and Methods

***Animals and experimental design***

All of the procedures involving the animals used in this study were carried out in accordance with the guidelines of the Institutional Animal Care and Use Committee of the Nanjing University of Chinese Medicine. The present study used male (SD) rats (320–350 g) purchased from the Zhao Yan (Suzhou) New Drug Research Center. A 12-hour light/dark cycle was employed in an environment with both a controlled temperature and a controlled humidity, and the rats had constant free access to both water and food.

In the first experiment, 42 out of 44 rats were divided randomly into seven groups, including a Sham group as well as a total of six SBI subgroups (these groups were 6 hr, 12 hr, 24 hr, 48 hr, 72 hr, and 7 d after SBI). At the specific time points specified above, the rats were euthanized **(**27**)** and their brain tissues from around the injured area were collected. The expression and localization of TFAM were determined via WB and IF, respectively.

In the second experiment, 48 rats (out of 49 total rats) were randomly divided into four groups, including a Sham group, an SBI group, an SBI+TMP (40 mg/kg), and an SBI + Vehicle group. There were twelve rats in each group. The rats were sacrificed after neurological scoring at 48 hr post-SBI. Brain tissue surrounding the injury was collected, and the expression levels of TFAM, Bcl-2, and Caspase-3 in their brain tissue were detected using WB. FJC and TUNEL staining were used to evaluate both necrosis and neuronal apoptosis, and the wet-dry method was employed to detect any brain edema. ROS and ATP levels were analyzed to assess mitochondrial function.

***Surgical brain injury rat model***

According to previous reports, the SBI rat model was established through an excision of a part of the right frontal lobe (28). The SD rats were anesthetized through the administration of an intraperitoneal injection containing 40 ml/kg sodium pentobarbital (29). After successful anesthesia, the rats were placed on a stereotaxic apparatus in the prone position (SA-100, Shanghai Yuyan Instruments, China). The skin on the top of the head was disinfected with Anerdian. The skin was cut, and the periosteum at the mid-sagittal line at the top of the skull was detached. A bone window with a diameter of about 5 mm was created at 2 mm before the right bregma and 2 mm beside the midline. The frontal lobe of the exposed area on the right side was removed. Once the bleeding stopped, the operation field was rinsed repeatedly with sterile normal saline until the flushing liquid was clear. The wound was disinfected and sutured. After the operation, the rats were closely monitored until they woke up after anesthesia.

***Drug injection***

TMP (40 mg/kg, MCE, USA) (30) dissolved in 10% DMSO + 90% corn oil was injected intraperitoneally 1 hr after SBI. The SBI + Vehicle group was given 10% DMSO + 90% corn oil at the same time point and dose.

***Western blot***

According to a previously published method (29), the rats’ brain tissues were completely disintegrated with RIPA lysis buffer and protease inhibitor (Beyotime, China). Then, the buffer mixture was centrifuged and the supernatant was collected. Using a PierceTM BCA Protein Assay Kit (Thermo Fisher, USA), the supernatant was aliquoted to determine the protein concentration, as well as the loading amount. The remaining supernatant was mixed with 5 X loading buffer (Beyotime, China) and boiled. The protein was isolated on SDS-polyacrylamide gels (Beyotime, China), and after that, it was then transferred to a PVDF membrane (Millipore, USA). This membrane was sealed at 37 °C for 1 hr using a QuickBlockTM Western (Beyotime, China) and incubated with primary antibody overnight at 4 °C. The antibodies used included rabbit anti-mtTFA (1:1000, Abcam, UK), rabbit anti-Bcl-2 (1:1000, Abcam, UK), rabbit anti-Caspase-3 (1:2000, Abcam, UK), and mouse anti-β-actin (1:1000, Sigma, USA) as a control group. Then, the primary antibody was recovered, and a sample was incubated with the corresponding secondary antibody, including goat anti-rabbit IgG-HRP (Invitrogen, USA) and goat anti-mouse IgG-HRP (Invitrogen USA). Then, a western chemiluminescent HRP substrate (Millipore, USA) and an imaging system (GE Healthcare Bio-Sciences AB, Sweden) were used to visualize the proteins, and the resulting bands were quantified using the ImageJ software.

***Immunofluorescent staining***

Immunofluorescent (IF) staining was performed according to a previously published method (31). Paraffin sections were first deparaffinized and then repaired with sodium citrate. An immunostaining permeable solution (Beyotime, China) was employed to penetrate the membrane. Tissue sections were then washed three times in PBS before being blocked with immunostaining blocking buffer (Beyotime, China) for 1 hr. After that, the tissues were incubated with both primary and secondary antibodies. Finally, DAPI anti-fluorescence quenching solution (YEASEN, China) was applied, and the tissues were observed using a fluorescence microscope after mounting (OLYMPUS, Japan). The antibodies used in the experiment included: rabbit anti-TFAM (Abcam, 1:500, UK), mouse anti-GFAP (Invitrogen, 1:1000, USA), goat anti-Iba1 (Abcam, 1:800, UK), mouse anti-NeuN (Abcam, 1:1000, UK), donkey anti-rabbit IgG, Alexa Fluor 488 (Invitrogen, 1:800, USA), donkey anti-mouse IgG, Alexa Fluor 555 (Invitrogen, 1:800, USA), and donkey anti-goat IgG.

***Brain water content***

The wet–dry method was employed to assess brain edema (32). The rats were first anesthetized and then they were decapitated. Their brains were then removed immediately and then divided into ipsilateral and contralateral sections. To determine the wet weight (WW), each section was weighed using an electronic analytical balance, and each was then subsequently oven-dried at 100 °C for 24 hr to determine the dry weight (DW). The brain water content was calculated as [(WW − DW)/WW] × 100%.

***Neurological score***

A modified Garcia score was used to assess and evaluate the rats’ neurological function, according to the methods used in a previous report (32). This modified score is composed of seven parameters: locomotion, body proprioception, tactile response, symmetry of limb movement, lateral rotation, forelimb walking, and climbing ability. Each parameter has a score of between 0 and 3, and the maximum score is thus 21. A high score is associated with a small degree of nerve damage.

***TUNEL staining***

TUNEL staining was used for the detection of neuronal apoptosis according to a previous method (33). The paraffin sections were deparaffinized and then incubated in proteinase K (37 °C for 20 min) and TUNEL detection liquid (Beyotime, China) (37 °C for 60 min). TUNEL-positive neurons were then detected with a U-RFL-T fluorescence microscope (OLYMPUS, Japan).

***Fluoro-jade C staining***

Neuronal necrosis was detected by FJC staining with a Biosensis kit (Biosensis, USA) (34). Paraffin sections were first deparaffinized, and then they were incubated in a potassium permanganate (at a concentration of 1:9 in distilled water) and FJC working solution (at a concentration of 1:10 in distilled water). The tissues were then rinsed with distilled water and dried at 60 °C for at least 5 min. The dried tissues were soaked in xylene for 1 min and fixed on a coverslip with stilbene plasticizer xylene (DPX). The FJC-positive cells were then imaged using a U-RFL-T fluorescence microscope (OLYMPUS, Japan).

***Measurement of ROS levels***

An ROS test kit (JianCheng, China) was used to measure the levels of ROS in brain tissues according to the method in a previously published study (35). Briefly, the samples were decomposed with 0.01 mol/lPBS and centrifuged at 4 °C and 500 × g for 10 min. Then, the supernatant (190 µl) and DCFH-DA (10 µl, 1 mol/l) were combined in micropores at room temperature for a total of 30 min. The mixture was then measured by fluorometry. A detergent-compatible protein assay kit (Bio-Rad, Hercules, CA, USA) was used to measure the concentration of protein; the ROS levels are expressed as fluorescent/mg protein.

***Measurement of ATP levels***

To quantify the ATP levels in the rats’ brain tissue, an ATP Assay Kit (Beyotime, Shanghai, China) was used. Then, 20 mg of brain tissue was combined with 200 µl of lysis solution and homogenized. The fully lysed tissues were centrifuged at 12000 g for 5 min at 4 ºC. ATP working reagent was added to the supernatant, and the absorbance was measured on a spectrophotometer (Thermo Fisher Scientific, USA). The sample’s ATP concentration was calculated according to the standard curve. A PierceTM BCA Protein Assay Kit (Thermo Fisher, USA) was also used to detect the protein concentration in the supernatant, and the ATP concentration was expressed as nmol/mg protein.

***Statistical analysis***

GraphPad Prism 8.0 software was used to perform statistical analyses, and all data are presented as the mean ± standard deviation. One-way analysis of variance (ANOVA) for multiple comparisons and the Student-Newman-Keuls *post hoc* test were used to determine the significant differences between the groups.* P*<0.05 was considered statistically significant.

## Results

***TFAM Expression after SBI***

The expression and localization of TFAM in nerve cells after SBI were assessed by determining the protein level of TFAM in the brain tissues around the injury area by WB. Compared to the Sham group, TFAM increased at 6 hr after SBI, peaked at 48 hr, and then gradually decreased to 7 d post-SBI (Figures 2a and b). TFAM was co-stained with the neuron marker NeuN, the astrocyte marker GFAP, or the microglial cell marker Iba1 by IF, and the results show TFAM was widely present in nerve cells. The number of TFAM-positive neurons (Figures 2c and d), astrocytes (Figures 2e and f), and microglia (Figures 2g and h) in the 48 hr post-SBI group increased, compared with those of the Sham group.

***Expression levels of TFAM, Bcl-2, and caspase-3 protein after TMP intervention***

To elucidate the mechanism of TFAM in nerve cells, rats were treated with TMP, and the expression of TFAM, Bcl-2, and Caspase-3 protein at 48 hr post-SBI was thus determined. TMP increased TFAM expression levels after SBI (Figures 3a and b). After TMP intervention, the expression of Bcl-2 increased (Figures 3c and d), while the expression of Caspase-3 decreased after TMP intervention (Figures 3e and f). Thus, the increase in TFAM can inhibit the apoptosis of nerve cells.

***TMP increased neurobehavioral function and decreased brain water content***

The modified Garcia test described above was used to assess neurological function, and the results show the neurobehavioral function of rats after SBI was worse than that of the Sham group, while the neurobehavioral function of rats improved after TMP intervention (Figure 4a). The wet-dry technique was also used to measure the water content of rat brains and evaluate the degree of brain edema. The results showed increased cerebral edema on the affected side after SBI and improved cerebral edema on the affected side after TMP intervention (Figure 4b).

***Effect of TMP intervention on ATP concentration and ROS concentration post-SBI***

ATP and ROS levels were measured to assess mitochondrial function. As shown in Figure 4c, ATP levels decreased after SBI but increased after TMP intervention. At the same time, ROS levels increased after SBI and decreased after TMP intervention (Figure 4d). These changes indicate improved mitochondrial function.

***Effect of TMP intervention on neuronal death after SBI***

TUNEL staining and FJC staining were used to assess the death of nerve cells. As expected, the death of nerve cells in the rat brain increased after SBI, but the death of nerve cells decreased after TMP intervention (Figure 5). Thus, TMP may improve mitochondrial function by increasing TFAM content, leading to increased ATP levels and decreased ROS levels, thereby inhibiting the Caspase-3 dependent apoptosis pathways. Ultimately, neuronal apoptosis decreased after SBI (Figure 6).

## Discussion

In this work, TFAM’s neuroprotective effect in an SBI rat model was evaluated, and the underlying mechanism for this effect was elucidated. Our experimental results show TFAM levels increased 6 hr after SBI, peaked at 48 hr, and then began to decline. TFAM was expressed in neurons, astrocytes, and microglia, and the expression of TFAM increased 48 hr after SBI. In addition, TMP intraperitoneal injection increased TFAM expression, which led to a decrease in ROS content and Caspase-3 expression, as well as an increase in ATP production and Bcl-2 expression. Moreover, TFAM can reduce brain water content, as well as neuronal apoptosis. These results suggest TFAM can improve cerebral edema and reduce neuronal apoptosis by increasing mitochondrial function, and we can infer that, in SBI, TFAM plays a protective role.

Mitochondria are organelles within cells that have multiple functions and are important regulatory factors of cellular energy metabolism and cellular redox balance (36). Previous studies have shown that when mitochondria dysfunction, the mitochondria-mediated apoptosis mechanism will be activated, resulting in irreversible damage to neurons (37-39). Therefore, the biogenesis that maintains mitochondrial function and activates mitochondria plays an important role in neuroprotection after brain injury. Our results showed that mitochondrial dysfunction induced by SBI can lead to the decrease of ATP level, the increase of ROS level, and the increase of Caspase-3 to increase apoptosis, and ultimately cause nerve damage. Relevant studies have shown that TFAM can directly regulate mtDNA abundance, and it plays an important role in maintaining mtDNA stability and mitochondrial biogenesis as well as cell response to energy demand. TFAM knockdown alone was sufficient to induce abnormal mtDNA nucleoid packaging and mitochondrial respiratory defects (40). 

TFAM, a member of the HMGB (high mobility box) subfamily, plays a key role in maintaining mtDNA (41), initiating mtDNA gene transcription, and protecting mitochondrial biogenesis from oxidative damage and ROS (42). Previous research has suggested that TFAM may increase the biogenesis of mitochondria after cerebral ischemia and hypoxia. Yin *et al*. measured TFAM levels 24 hr after hypoxic-ischemic brain injury (43). TFAM levels increased, and mtDNA levels also increased. In addition, the authors found a time-dependent increase in mitochondrial number, in the expression levels of the mitochondrial proteins of HSP60 and COXIV, and citrate synthase activity. Based on these results, after hypoxic-ischemic brain injury, an increase in mitochondrial biogenesis can be inferred. Due to this increase in mitochondrial mass, the overall energy status and oxidation function of the H-I brain significantly improved, which may be an endogenous neuroprotective response to H-I injury. However, previous experiments only detected the changes in TFAM levels at 24 hr post-hypoxic-ischemic brain injury. Our experiment began 6 hr post-SBI and lasted for 7 d. During this time, TFAM increased at 6 hr after SBI and peaked at 48 hr, which is consistent with previous studies. The level of TFAM decreased gradually after 48 hr. In addition, we also detected the levels of ATP, ROS, and apoptosis at 48 hr after SBI. Despite the higher level of TFAM after 48 hr in the SBI 48 hr group, compared with the Sham group, the level of ATP was lower. In addition, the ROS level was higher in this group than in the Sham group, and the apoptosis rate of the 48 hr group was also higher than that of the Sham group. This result is in agreement with previous research, which showed that mitochondrial biogenesis occurs rapidly after brain injury as an endogenous neuroprotective response of the body. However, this endogenous protective response is limited and can only delay the onset of damage. With the ischemia and hypoxia of local brain tissue after brain injury, mitochondrial dysfunction, nerve cell apoptosis, and nerve function injury may occur.

The use of exogenous interventions can increase TFAM levels to further increase mitochondrial biogenesis and alleviate mitochondrial dysfunction. TFAM therapies would thereby increase ATP levels, reduce ROS production, and mitigate neuronal apoptosis. TMP can specifically block the Lon protease-mediated degradation of TFAM. For cells with significantly lower mtDNA levels, this leads to TFAM accumulation and the subsequent up-regulation of mtDNA. The work presented here also verified this result. In this study, the level of TFAM in rat nerve cells increased upon intraperitoneal injection of TMP. The level of TFAM in the SBI + TMP group was higher than that in the SBI + Vehicle group. At the same time, we tested two levels of ATP and ROS in the group as well as the protein levels of Caspase-3 and Bcl-2. Rats that received TMP intervention had better mitochondrial function and lower levels of cell apoptosis, compared with the control.

In summary, exogenously increasing TFAM levels can delay mitochondrial dysfunction, inhibit neuronal apoptosis, and ultimately reduce secondary brain damage after SBI. Therefore, the mitochondrial pathway involved in TFAM may be an important, endogenous, and physiological regulation-signaling pathway in neurons, and it can be used as a therapeutic target to reduce post-SBI brain injury. Despite these important findings, our research had limitations. For example, the level of TFAM was not down-regulated to study whether down-regulating TFAM would increase the damage associated with mitochondrial function, which would then subsequently increase post-SBI neuronal damage, thereby also increasing nerve function damage. We will further verify this in future research.

**Figure 1:**
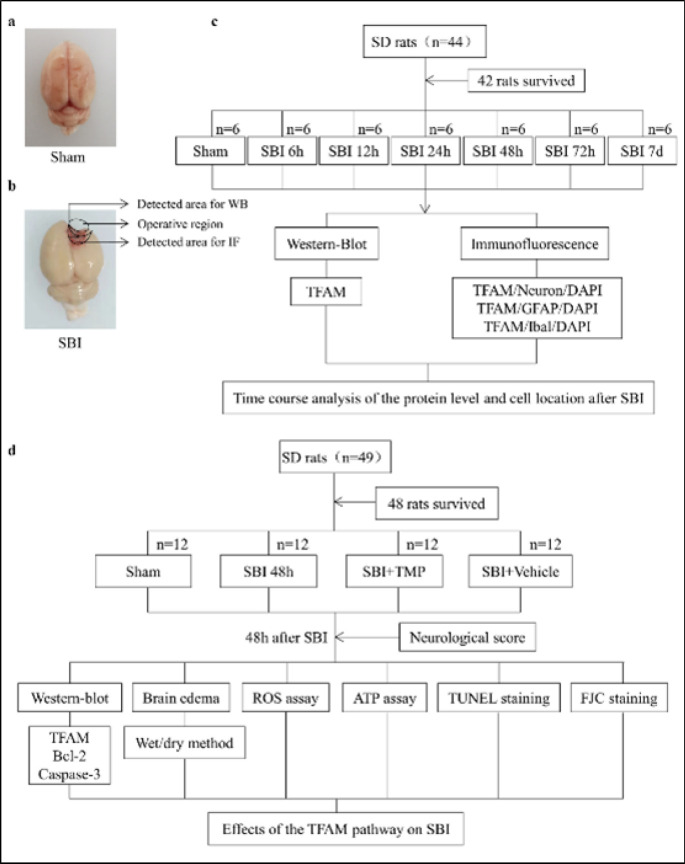
Their corresponding locations in the Sham group in rats (a), and images of brain tissue around the brain injury in the SBI group in rats (b) and the expression and localization of TFAM in nerve cells after SBI in rats (c) and the influence of TMP on nerve function after SBI in rats (d)

TFAM: Mitochondrial transcription factor A; SBI: Surgical brain injury; TMP: Tetramethylpyrazine

**Figure 2:**
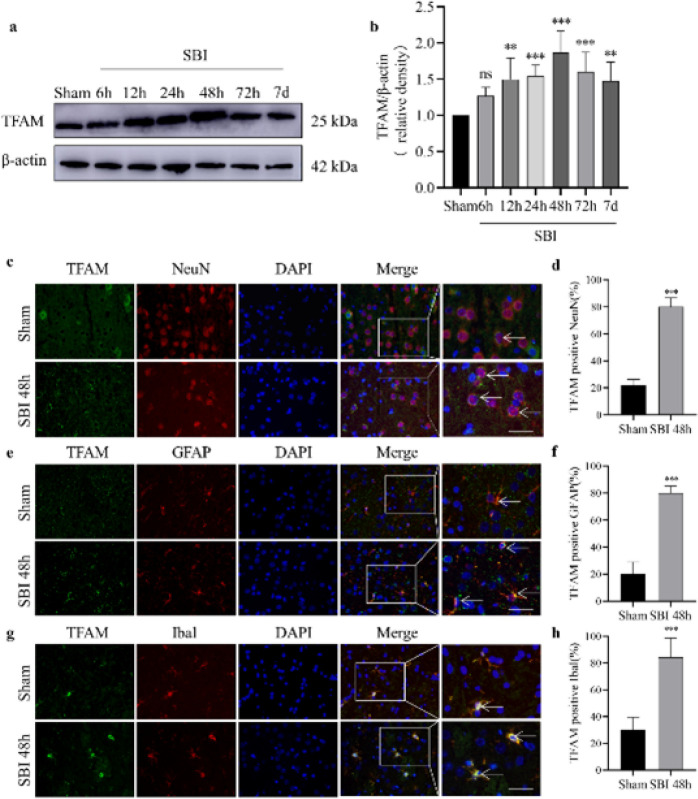
Expression and localization of TFAM in the brain tissue around the injury after SBI in rats

TFAM protein expression levels in the Sham (a) and SBI (b) groups at 6 hr, 12 hr, 24 hr, 48 hr, 72 hr, and 7 d, as determined by WB. Protein expression levels were quantified via Image J software. The protein expression of the Sham group was averaged and normalized to 1, and the relative density of each protein was normalized to the Sham group. One-way ANOVA was used for statistical analysis, followed by the Student–Newman–Keuls post hoc test, n = 6 for each group. Data are expressed as the mean ± SD. ** *P*<0.01, *** *P*<0.001, ns *P*>0.05, compared to the Sham group. In the Sham group and 48 hr after SBI, IF was used to detect the expression of green-labeled TFAM and red-labeled NeuN (c, d), GFAP (e, f), and Iba1 (g, h). The nucleus was fluorescently labeled with DAPI (blue). The arrow indicates the co-localization of TFAM with neurons, astrocytes, and microglia. Scale bar = 50 µm. Student's t-test (n = 6) was used for statistical analysis. Data are expressed as the mean ± SD. *** *P*<0.001, compared with the Sham group.

TFAM: Mitochondrial transcription factor A; SBI: Surgical brain injury

**Figure 3:**
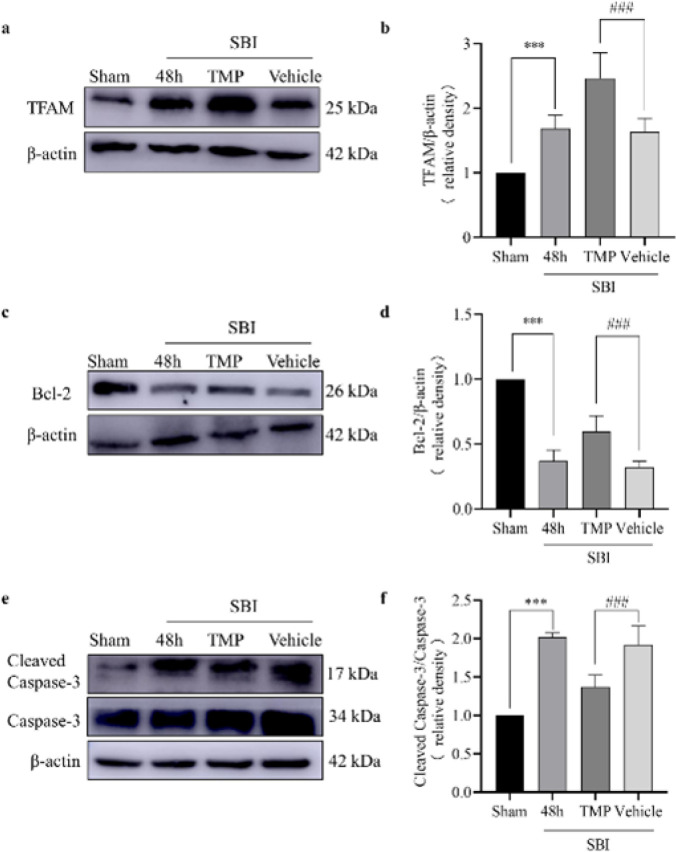
Effect of TMP intervention on the expression level of related proteins 48 h after SBI in rats

The protein expression levels of TFAM (a, b), Bcl-2 (c, d), and Caspase-3 (e, f) were measured, and the effect of TMP intervention on brain injury 48 hr after SBI was evaluated. The statistical analysis was performed using one-way ANOVA, and then the Student–Newman–Keuls post hoc test was used. Data are expressed as the mean ± SD. n = 6 for each group and Sham group. *** *P*<0.001, compared with the Sham group. ### *P*<0.001, compared with SBI + Vehicle group.

TFAM: Mitochondrial transcription factor A; SBI: Surgical brain injury

**Figure 4:**
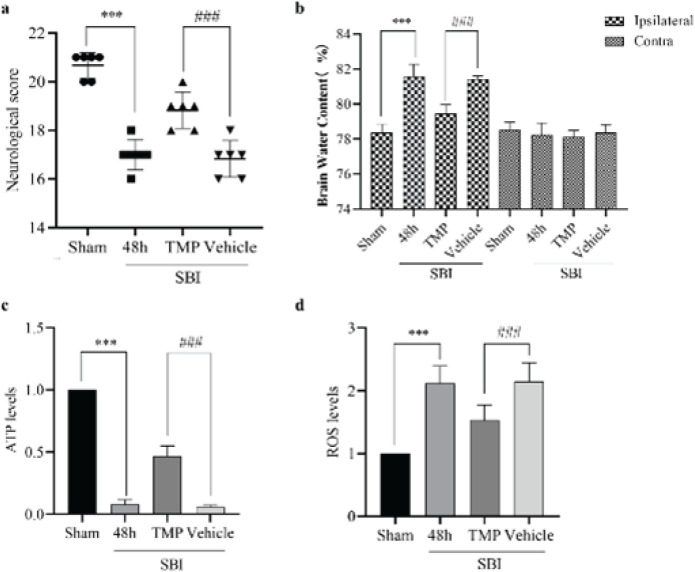
Effect of TMP intervention on the neurological score, cerebral edema, and the concentrations of ATP and ROS 48 hr after SBI in rats

The neurobehavioral score of SBI rats after TMP intervention (a). The brain water content of the bilateral hemispheres in different groups, as determined by the wet-dry method (b). ATP (c) and ROS (d) levels, which were used to evaluate the effect of TMP intervention on nerve injury 48 hr after SBI. The statistical analysis was performed using one-way ANOVA, and the Student–Newman–Keuls *post hoc *test was used. Data are expressed as the mean ± SD. n = 6 in each group. *** *P*<0.001, compared with the Sham group. ### *P*<0.001, compared with SBI + Vehicle group.

ROS: Reactive oxygen species; TMP: Tetramethylpyrazine; SBI: Surgical brain injury; ATP: Adenosine triphosphate

**Figure 5:**
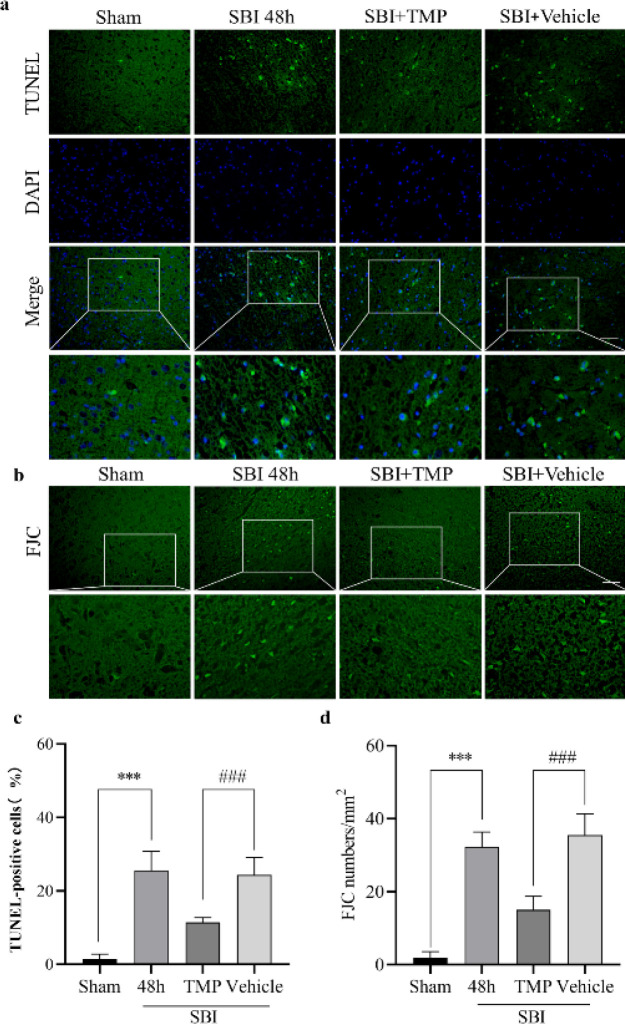
Effects of TMP intervention on nerve cells 48 hr after SBI in rats

Neuronal apoptosis was determined via TUNEL staining (a, c). TUNEL (green) staining indicated cell apoptosis and DAPI (blue) marked cell nuclei. FJC (green) staining was used to detect nerve cell necrosis (b, d). Scale bar = 50 µm. Statistical analysis was performed using one-way ANOVA, followed by the Student–Newman–Keuls *post hoc* test. Data are expressed as the mean ± SD. n = 6 for each group. *** *P*<0.001, compared with the Sham group. ### *P*<0.001 compared with SBI + Vehicle group

TMP: Tetramethylpyrazine; SBI: Surgical brain injury

**Figure 6:**
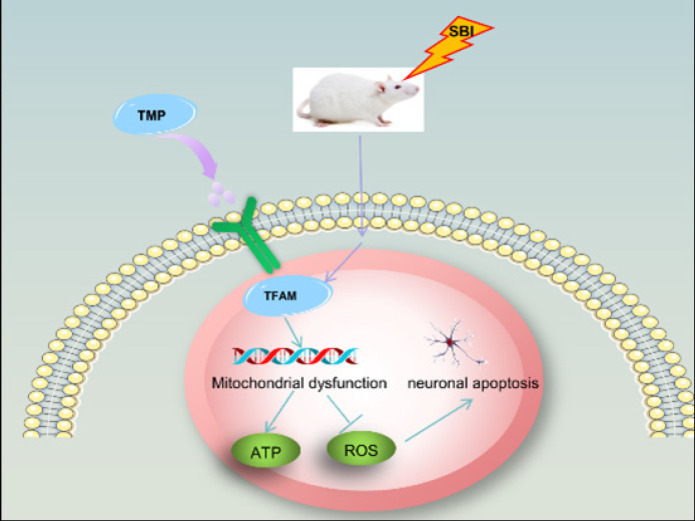
Mechanism driving TMP regulation of secondary brain injury post-SBI in rats

After SBI, TMP increased the expression of TFAM in nerve cells, mitigated mitochondrial dysfunction, increased ATP levels, and decreased ROS levels, to reduce mitochondrial dysfunction and apoptosis.

TMP: Tetramethylpyrazine; TFAM: Mitochondrial transcription factor A; SBI: Surgical brain injury; ROS: Reactive oxygen species

## Conclusion

In this study, it was found that after SBI, treatment with TMP increased TFAM levels, promoted mitochondrial function, reduced ROS production, and increased ATP levels. As a result, Caspase-3 was inhibited and Bcl-2 levels increased, thereby reducing brain edema and nerve cell apoptosis. Thus, we concluded that TMP played a role in brain protection. As such, TFAM could be an important future target for both preventing and treating secondary brain injury after SBI.

## Authors’ Contributions

B D contributed to the study’s conception and design. M W, Y G, Y H, and M Z performed material preparation, and data collection and analysis. C W wrote the first draft of the manuscript, and all authors commented on previous versions of the manuscript. All authors read and approved the final manuscript.

## Declaration

The authors confirm that no paper mill was used and that all data were generated in-house.

## Ethics Approval

The animal study was reviewed and approved by the Institute of Animal Care Committee of Zhangjiagang Traditional Chinese Medicine Hospital.

## Availability of Data and Materials

Please contact the corresponding author.

## Conflicts of Interest

The authors have no relevant financial or non-financial interests to disclose.

## References

[1] Dewan M C, Rattani A, Fieggen G, Arraez M A, Servadei F, Boop F A (2018). Global neurosurgery: The current capacity and deficit in the provision of essential neurosurgical care Executive summary of the global neurosurgery initiative at the program in global surgery and social change. J Neurosurg.

[2] Sherchan P, Huang L, Akyol O, Reis C, Tang J, Zhang J H (2017). Recombinant Slit2 reduces surgical brain injury induced blood brain barrier disruption via Robo4 dependent rac1 activation in a rodent model. Sci Rep.

[3] Akyol O, Sherchan P, Yilmaz G, Reis C, Ho W M, Wang Y (2018). Neurotrophin-3 provides neuroprotection via TrkC receptor dependent pErk5 activation in a rat surgical brain injury model. Exp Neurol.

[4] Kim C H, McBride D W, Sherchan P, Person C E, Gren E C K, Kelln W (2017). Crotalus helleri venom preconditioning reduces postoperative cerebral edema and improves neurological outcomes after surgical brain injury. Neurobiol Dis.

[5] Xiao Y, Li G, Chen Y, Zuo Y, Rashid K, He T (2018). Milk fat globule-epidermal growth factor-8 pretreatment attenuates apoptosis and inflammation via the integrin-beta3 pathway after surgical brain injury in rats. Front Neurol.

[6] Chen Q, Xu L, Du T, Hou Y, Fan W, Wu Q (2019). Enhanced expression of pd-l1 on microglia after surgical brain injury exerts self-protection from inflammation and promotes neurological repair. Neurochem Res.

[7] Jeong S Y, Seol D W (2008). The role of mitochondria in apoptosis. BMB Rep.

[8] Prentice H, Modi J P, Wu J Y (2015). Mechanisms of neuronal protection against excitotoxicity, endoplasmic reticulum stress, and mitochondrial dysfunction in stroke and neurodegenerative diseases. Oxid Med Cell Longev.

[9] Misgeld T, Schwarz T L (2017). Mitostasis in neurons: Maintaining mitochondria in an extended cellular architecture. Neuron.

[10] Fan H, Ding R, Liu W, Zhang X, Li R, Wei B (2021). Heat shock protein 22 modulates NRF1/TFAM-dependent mitochondrial biogenesis and DRP1-sparked mitochondrial apoptosis through AMPK-PGC1alpha signaling pathway to alleviate the early brain injury of subarachnoid hemorrhage in rats. Redox Biol.

[11] Liu C, Ma J, Zhang J, Zhao H, Zhu Y, Qi J (2019). Testosterone deficiency caused by castration modulates mitochondrial biogenesis through the ar/pgc1alpha/tfam pathway. Front Genet.

[12] Yu J, Zheng J, Lu J, Sun Z, Wang Z, Zhang J (2019). AdipoRon protects against secondary brain injury after intracerebral hemorrhage via alleviating mitochondrial dysfunction: Possible involvement of AdipoR1-AMPK-PGC1alpha pathway. Neurochem Res.

[13] Ulbrich F, Kaufmann K B, Meske A, Lagreze W A, Augustynik M, Buerkle H (2016). The CORM ALF-186 mediates anti-apoptotic signaling via an activation of the p38 MAPK after ischemia and reperfusion injury in retinal ganglion cells. PLoS One.

[14] Park S, Yamaguchi M, Zhou C, Calvert J W, Tang J, Zhang J H (2004). Neurovascular protection reduces early brain injury after subarachnoid hemorrhage. Stroke.

[15] Sorrentino V, Menzies K J, Auwerx J (2018). Repairing mitochondrial dysfunction in disease. Annu Rev Pharmacol Toxicol.

[16] Rizwan H, Pal S, Sabnam S, Pal A (2020). High glucose augments ROS generation regulates mitochondrial dysfunction and apoptosis via stress signalling cascades in keratinocytes. Life Sci.

[17] Wei W, Wang H, Wu Y, Ding K, Li T, Cong Z (2015). Alpha lipoic acid inhibits neural apoptosis via a mitochondrial pathway in rats following traumatic brain injury. Neurochem Int.

[18] Huang J L, Manaenko A, Ye Z H, Sun X J, Hu Q (2016). Hypoxia therapy--a new hope for the treatment of mitochondrial dysfunctions. Med Gas Res.

[19] Zhou J, Yang Z, Shen R, Zhong W, Zheng H, Chen Z (2021). Resveratrol improves mitochondrial biogenesis function and activates PGC-1alpha pathway in a preclinical model of early brain injury following subarachnoid hemorrhage. Front Mol Biosci.

[20] Picca A, Pesce V, Fracasso F, Joseph A M, Leeuwenburgh C, Lezza A M (2014). A comparison among the tissue-specific effects of aging and calorie restriction on TFAM amount and TFAM-binding activity to mtDNA in rat. Biochim Biophys Acta.

[21] Kunkel G H, Chaturvedi P, Tyagi S C (2016). Mitochondrial pathways to cardiac recovery: TFAM. Heart Fail Rev.

[22] Matsushima Y, Goto Y, Kaguni L S (2010). Mitochondrial Lon protease regulates mitochondrial DNA copy number and transcription by selective degradation of mitochondrial transcription factor A (TFAM). Proc Natl Acad Sci U S A.

[23] Kasashima K, Endo H (2015). Interaction of human mitochondrial transcription factor A in mitochondria: Its involvement in the dynamics of mitochondrial DNA nucleoids. Genes Cells.

[24] Huang B, You J, Qiao Y, Wu Z, Liu D, Yin D (2018). Tetramethylpyrazine attenuates lipopolysaccharide-induced cardiomyocyte injury via improving mitochondrial function mediated by 14-3-3gamma. Eur J Pharmacol.

[25] Li S, Xiao X, Ni X, Ye Z, Zhao J, Hang C (2017). Tetramethylpyrazine protects against early brain injury after experimental subarachnoid hemorrhage by affecting mitochondrial-dependent caspase-3 apoptotic pathway. Evid Based Complement Alternat Med.

[26] Lan L, Guo M, Ai Y, Chen F, Zhang Y, Xia L (2017). Tetramethylpyrazine blocks TFAM degradation and up-regulates mitochondrial DNA copy number by interacting with TFAM. Biosci Rep.

[27] Zatroch K K, Knight C G, Reimer J N, Pang D S J (2017). Refinement of intraperitoneal injection of sodium pentobarbital for euthanasia in laboratory rats (Rattus norvegicus). BMC Veterinary Research.

[28] Gong Y, Wu M, Gao F, Shi M, Gu H, Gao R (2021). Inhibition of the pSPAK/pNKCC1 signaling pathway protects the bloodbrain barrier and reduces neuronal apoptosis in a rat model of surgical brain injury. Mol Med Rep.

[29] Gong Y, Wu M, Shen J, Tang J, Li J, Xu J (2021). Inhibition of the NKCC1/NF-kappaB signaling pathway decreases inflammation and improves brain edema and nerve cell apoptosis in an SBI rat model. Front Mol Neurosci.

[30] Gong P, Zhang Z, Zou Y, Tian Q, Han S, Xu Z (2019). Tetramethylpyrazine attenuates blood-brain barrier disruption in ischemia/reperfusion injury through the JAK/STAT signaling pathway. Eur J Clin Pharmacol.

[31] Shi M, Gong Y, Wu M, Gu H, Yu J, Gao F (2022). Downregulation of TREM2/NF-small ka, CyrillicB signaling may damage the blood-brain barrier and aggravate neuronal apoptosis in experimental rats with surgically injured brain. Brain Res Bull.

[32] Wu M Y, Gao F, Yang X M, Qin X, Chen G Z, Li D (2020). Matrix metalloproteinase-9 regulates the blood brain barrier via the hedgehog pathway in a rat model of traumatic brain injury. Brain Res.

[33] Meng C, Zhang J, Dang B, Li H, Shen H, Li X (2018). PERK pathway activation promotes intracerebral hemorrhage induced secondary brain injury by inducing neuronal apoptosis both in vivo and in vitro. Front Neurosci.

[34] Wu M Y, Gao F, Tang J F, Shen J C, Gao R, Dang B Q (2021). Possible mechanisms of the PERK pathway on neuronal apoptosis in a rat model of surgical brain injury. Am J Transl Res.

[35] Zheng J, Shi L, Liang F, Xu W, Li T, Gao L (2018). Sirt3 ameliorates oxidative stress and mitochondrial dysfunction after intracerebral hemorrhage in diabetic rats. Front Neurosci.

[36] Bhatti J S, Bhatti G K, Reddy P H (2017). Mitochondrial dysfunction and oxidative stress in metabolic disorders - A step towards mitochondria based therapeutic strategies. Biochim Biophys Acta Mol Basis Dis.

[37] Owens K, Park J H, Schuh R, Kristian T (2013). Mitochondrial dysfunction and NAD(+) metabolism alterations in the pathophysiology of acute brain injury. Transl Stroke Res.

[38] Kumar Sahel D, Kaira M, Raj K, Sharma S, Singh S (2019). Mitochondrial dysfunctioning and neuroinflammation: Recent highlights on the possible mechanisms involved in traumatic brain injury. Neurosci Lett.

[39] Wu Y, Chen M, Jiang J (2019). Mitochondrial dysfunction in neurodegenerative diseases and drug targets via apoptotic signaling. Mitochondrion.

[40] Zhao M, Wang Y, Li L, Liu S, Wang C, Yuan Y (2021). Mitochondrial ROS promote mitochondrial dysfunction and inflammation in ischemic acute kidney injury by disrupting TFAM-mediated mtDNA maintenance. Theranostics.

[41] Campbell C T, Kolesar J E, Kaufman B A (2012). Mitochondrial transcription factor A regulates mitochondrial transcription initiation, DNA packaging, and genome copy number. Biochim Biophys Acta.

[42] Theilen N T, Kunkel G H, Tyagi S C (2017). The role of exercise and tfam in preventing skeletal muscle atrophy. J Cell Physiol.

[43] Yin W, Signore A P, Iwai M, Cao G, Gao Y, Chen J (2008). Rapidly increased neuronal mitochondrial biogenesis after hypoxic-ischemic brain injury. Stroke.

